# Oligoester Identification in the Inner Coatings of Metallic Cans by High-Pressure Liquid Chromatography–Mass Spectrometry with Cone Voltage-Induced Fragmentation

**DOI:** 10.3390/ma17112771

**Published:** 2024-06-06

**Authors:** Monika Beszterda-Buszczak, Rafał Frański

**Affiliations:** 1 Department of Food Biochemistry and Analysis, Poznań University of Life Sciences, Mazowiecka 48, 60-623 Poznań, Poland; monika.beszterda@gmail.com; 2 Faculty of Chemistry, Adam Mickiewicz University, Uniwersytetu Poznańskiego 8, 61-614 Poznań, Poland

**Keywords:** can-coating material, polyesters, mass spectrometry, fragmentation pathway, liquid chromatography

## Abstract

The application of polyesters as food contact materials is an alternative to epoxy resin coatings, which can be a source of endocrine migrants. By using high-pressure liquid chromatography/electrospray ionization–mass spectrometry (HPLC/ESI-MS) with cone voltage-induced fragmentation in-source, a number of polyester-derived migrants were detected in the extracts of inner coatings of metallic cans. The polyester-derived migrants were detected in each inner coating of fish product-containing cans (5/5) and in one inner coating of meat product-containing can (1/5). They were not detected in the inner coatings of vegetable/fruit product-containing cans (10 samples). The respective detected parent and product ions enabled differentiation between cyclic and linear compounds, as well as unambiguous identification of diol and diacid units. Most of the detected compounds, cyclic and linear, were composed of neopentyl glycol as diol and two diacid comonomers, namely isophthalic acid and hexahydrophthalic acid. The other detected oligoesters were composed of neopentyl glycol or propylene glycol and adipic acid/isophthalic acid as comonomers. The compounds containing propylene glycol as diol were found to be exclusively linear cooligoesters. On the basis of abundances of [M+Na]^+^ ions, the relative contents of cyclic and linear oligoesters were evaluated.

## 1. Introduction

Polyesters are a very common class of polymers with a number of industrial and everyday life applications, e.g., in car manufacturing, clothing, innovative materials for biomedical applications, and food packaging materials [1,2,3,4,5,6]. The latter application, as food contact materials, may be regarded as an alternative to epoxy resin coatings, which are a source of endocrine migrants (e.g., bisphenol A diglycidyl ether conjugates [7]). On the other hand, polyester-based food contact materials are relatively prone to being hydrolyzed and may also be a source of undesired migrants and thus non-intentionally added substances [8,9]. The other sources of polyester-derived migrants may be polyester thermal degradation, incomplete polymerization, or the formation of by-products during the manufacturing process. The determination of the structure of this kind of migrant is an area of research that has been recently widely developed in the field of food packaging materials and non-intentionally added substances; however, the reported data are definitely less numerous than those devoted to epoxy resin-derived migrants and requires further exploration. The vast majority of the reported polyester-derived migrants are cyclic oligoesters [10,11,12,13,14,15,16,17,18,19,20,21,22,23]. The formation of cyclic oligoesters in higher amounts in comparison to their linear counterparts may be related to the conditions of the synthesis process, e.g., under high dilution conditions, intramolecular esterification may be statistically favored [13,24]. Furthermore, linear oligoesters are less stable and more reactive than cyclic oligoesters and may be covalently bound to the food contact material [14].

Since, in order to prepare the polyesters, various diols and diacids are used (including isomeric monomers, e.g., isophthalic/terephthalic acids), a vast number of polyester-derived migrant structures have to be considered, including co-oligomers, for which the respective standards may be unavailable [5,24,25]. Thus, the elucidation of their structures may be a challenge. Therefore, the tentative identification of the oligoesters detected is often acceptable [10,14,17,25,26,27,28]. On the other hand, there are a number of papers reporting that detailed mass spectrometric analysis provided valuable structural data, which permitted their identification to be as justified as possible. For low-molecular-weight oligoesters, which are sufficiently volatile, GC-MS has been successfully used since the obtained EI mass spectra are always rich in the peaks of fragment ions, which well reflect the structure of parent compounds [10,13,15]. However, the identification of most oligoesters requires the use of HPLC-MS analysis. There are very valuable examples of detailed MS/MS data obtained from HPLC-MS/MS analysis. The data enabled the determination of the oligoester fragmentation patterns and, as a consequence, permitted the verification of the proposed structures [10,14,18,20,28]. The most comprehensive and detailed MS/MS data on polyester-derived migrants have been reported by Cariou et al. [29]. The conditions applied by the authors allowed for avoiding the fragmentation in-source; therefore, besides typical ions as [M+H]^+^, [M+NH_4_]^+^, and [M+Na]^+^, acetonitrile-containing adducts [M+NH_4_+CH_3_CN]^+^ and others were detected as well, which was of high analytical value [29].

In this work, we applied HPLC-MS analysis with cone voltage-induced fragmentation in-source (collision-induced dissociation in-source, CID in-source) in order to identify the most common classes of polyester-derived potential migrants present in the can-coating materials marketed in Poland. Due to well-known limitations [30,31], cone voltage-induced fragmentation in-source, although long recognized, is definitely less utilized than collision-induced dissociation–tandem mass spectrometry (CID-MS/MS). On the other hand, there are well-documented examples showing that cone voltage-induced fragmentation in-source enabled detailed structure elucidation [32,33,34,35,36,37,38]. This technique does not require the selection of precursor ions (which on untargeted analysis of complex mixtures may be a challenge) and allows the use of less expensive instruments than those used for CID-MS/MS experiments. To the best of our knowledge, the application of HPLC-MS with cone voltage-induced fragmentation in-source for the structure elucidation of polyester-derived migrants has not been reported yet.

## 2. Materials and Methods

A total number of 20 canned food samples were collected from supermarkets in Western Poland in January 2024 (description shown in the Supplementary Materials, Table S1). All the canned food products were collected in duplicate and initially stored in their original containers without exceeding the expiration date. The empty cans with a net weight from 100 to 170 g were filled with 50 mL of acetonitrile, while the cans with a net weight from 300 to 400 g and from 700 to 800 g were filled with 150 and 300 mL of acetonitrile, respectively. The mechanical extraction process was performed by carefully stirring the empty cans filled with an appropriate volume of acetonitrile. Open cans were attached to a shaker, and their contents were gently mixed in a reciprocating motion for 45 min. The extraction procedure was performed twice. The extracts were concentrated to a minimum by evaporation in a vacuum, and the residues were re-dissolved to a final volume of 3 mL with acetonitrile. Prior to the HPLC-MS analysis, the sample was further filtered through syringe filters with a pore size of 0.45 µm.

The HPLC-MS analyses were made on a Waters model 2690 HPLC pump (Milford, MA, USA) and Waters/Micromass ZQ2000 mass spectrometer (a single quadrupole-type instrument equipped with an electrospray ion (ESI) source, Z-spray, Manchester, UK). The software used was MassLynx V3.5 (Manchester, UK). The sample solutions were injected into the XBridge^®^ C18 column (3.5 µm, 100 mm × 3 mm i.d.; Waters, Warsaw, Poland) using an autosampler. The injection volume was 10 µL. The solutions were analyzed using a linear gradient of CH_3_CN-H_2_O (the most common type of gradient used for oligoester analysis, e.g., [8,10,29]), with a flow rate of 0.4 mL/min. The gradient started from 0% CH_3_CN to 95% H_2_O with 5% of a 10% solution of formic acid in water (in other words, the 10% solution of HCOOH was continuously dosed with 5% of the total flow rate), reaching 95% CH_3_CN after 25 min, and the latter concentration was kept for 15 min. The HPLC-MS was performed in the positive ion mode in the *m*/*z* range 100–1200. Each sample was analyzed twice. The nebulizing and desolvation gases were nitrogen at the flow rates of 100 and 300 L/h, respectively. The source temperature was 120 °C, and the desolvation temperature was 300 °C. The electrospray source potentials were capillary 3 kV, lens 0.5 V, extractor 4 V, and cone voltage (CV) was 30 and 50 V; the latter higher value enabled detection of the important diagnostic product ions.

## 3. Results and Discussion

The polyester-derived migrants were detected in the extracts of inner coatings of fish products-containing cans (samples 16 CF, 17 CF, 18 CF, 19 CF, 20 CF) and in one inner coating of meat product-containing can (sample 11 CM). This means that polyester material is nowadays mainly used as a food contact material in fish cans, and in other food cans, different types of inner coatings are used, e.g., epoxy resin. Most of the detected polyester-derived migrants can be described as cooligoesters, cyclic and linear, consisting of one diol monomer, namely neopentyl glycol (NPG), and two diacid comonomers, namely isophthalic acid/hexahydrophthalic acid (iPA/HHA). In one sample (20 CF), we detected the NPG cooligoesters, cyclic and linear, containing adipic acid/isophthalic acid (AA/iPA) as comonomers. In one sample (11 CM), we detected linear cooligoesters consisting of propylene glycol (1,2-propanodiol, PD) and AA/iPA as comonomers.

**Scheme 1:**
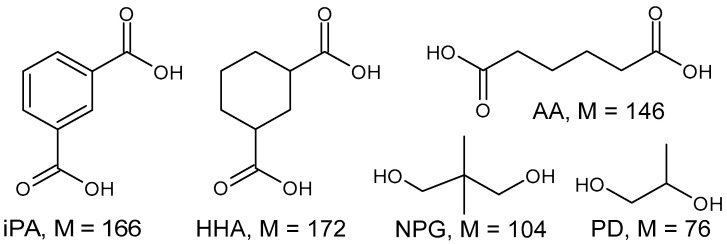
Plausible structures, abbreviations, and masses of the diacids and diols from which the detected oligoesters are composed.

Scheme 1 shows the plausible monomer structures; however, it is clear that their isomers cannot be excluded, and the detected oligoesters can contain a mixture of isomeric monomers (e.g., isophthalic acid/terephthalic acid; 1,2-propanediol/1,3-propanediol). Furthermore, the presence of sequential isomers, which have similar retention times, is also very likely. The detected potential polyester-derived migrants are listed in Table 1, Table 2 and Table 3.

**Figure 1:**
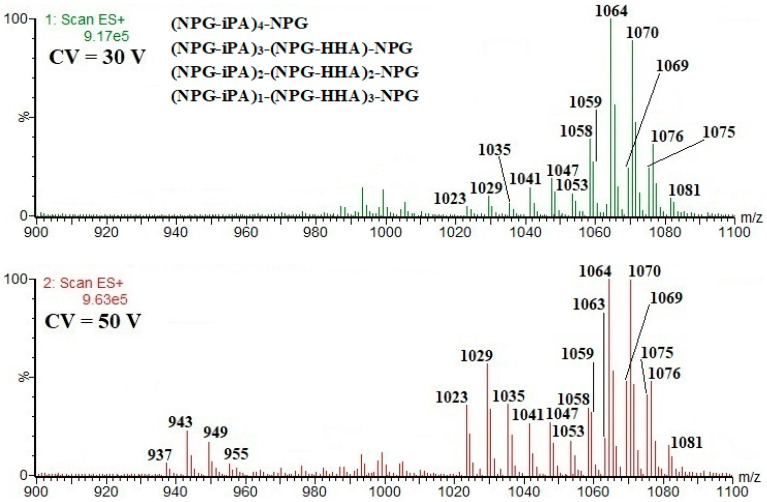
ESI mass spectra of linear (NPG-iPA/HHA)_n_-NPG oligoesters obtained for sample 17 CF (see Table 1 for ion assignments).

**Figure 2:**
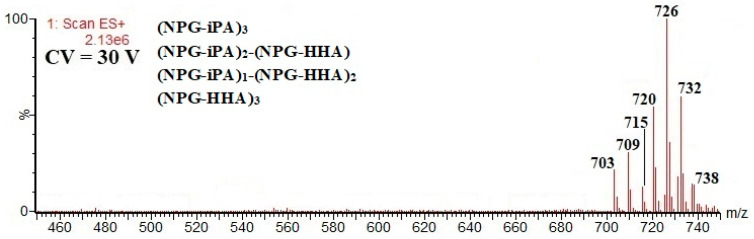

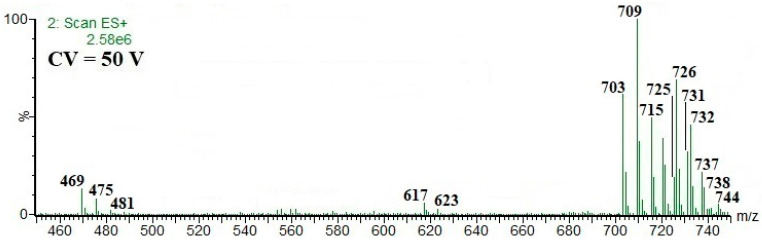
ESI mass spectra of cyclic (NPG-iPA/HHA)_n_ oligoesters obtained for sample 17 CF (see Table 3 for ion assignments).

**Figure 3:**
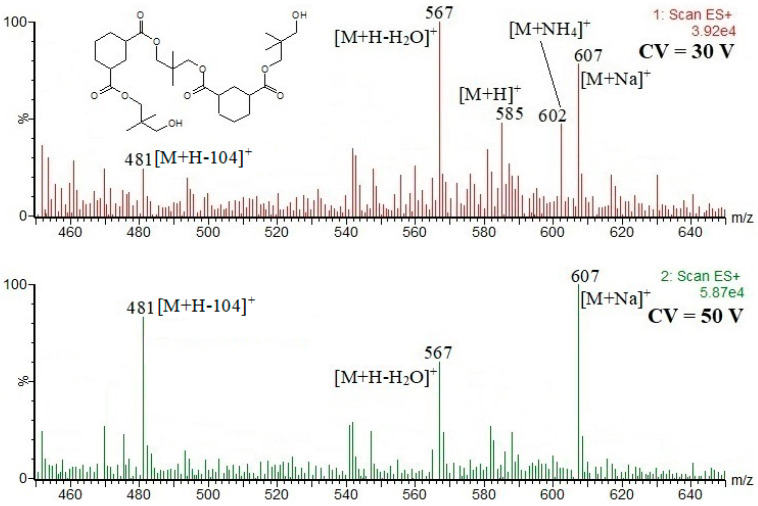
ESI mass spectra of linear (NPG-HHA)_2_-NPG oligoester obtained for sample 17 CF and its plausible structure.

The iPA/HHA-containing cooligoesters (the most common ones) were detected as a series of ions differentiated by six Daltons, and the respective oligomers were hardly separated chromatographically (for example, see Supplementary Materials, Figures S1 and S2). Therefore, the obtained ESI spectra were often quite complicated, as shown in Figure 1 and Figure 2, although their careful inspection enabled all ions assignments. By taking the ESI spectra from the edge of the chromatographic peaks, it was sometimes possible to obtain relatively pure mass spectra; an example is shown in Figure 3 for the linear (NPG-HHA)_2_-NPG.

The AA/iPA-containing cooligoesters were detected as a series of ions (peaks) differentiated by twenty Daltons, and the respective oligomers were better separated chromatographically than iPA/HHA-containing cooligoesters (for example, see Supplementary Materials, Figures S3 and S4). Therefore, for most of them, it was possible to obtain relatively pure mass spectra (for example, see Supplementary Materials, Figures S5–S8).

The characteristic two features of linear oligoester fragmentation, which distinguish them from cyclic oligoesters, are the loss of water molecule and the loss of diol molecule (loss of mass 104 for NPG conjugates and loss of mass 76 for PD conjugates). Rhe loss of the diol molecule, which is observed at a higher CV value, is especially of importance with respect to the structure elucidation of the unknown compounds. Of course, all ions derived from a given compound yielded respective chromatographic peaks at identical retention times; a representative example for (NPG-AA)_3_-(NPG-iPA)-NPG is shown in the Supplementary Materials (Figure S9). The characteristic features of cyclic oligoester fragmentation are the loss of dehydrated neopentyl glycol molecule (loss of mass 86), the loss of NPG-iPA unit (loss of mass 234), and/or the loss of NPG-AA unit (loss of mass 214). The loss of NPG-HHA (loss of mass 240) cannot be definitively excluded. However, we found that the oligoesters containing more HHA units dissociate to a lesser extent than those containing more iPA. Although iPA/HHA exchange causes almost negligible change in the mass, it substantially increases the number of degrees of freedom, which is defined as DoF = 3N − 6 (N stands for the number of atoms in the ion). The conversion of collision energy into vibrational energy is of crucial importance for the gas-phase ion dissociation process, and this conversion depends on the DoF of the ion subjected to the CID process [39,40]. It explains why the oligoesters containing more HHA units dissociate to a lesser extent than those containing more iPA. The AA/iPA exchange slightly decreases the DoF and slightly increases the mass, and these changes have the opposite effects on the gas-phase ion dissociation process (heavier ions dissociate to a lesser extent).

It must be pointed out that the linear cooligoesters consisted of propylene glycol (1,2-propanodiol, PD), and AA/iPA (detected in sample 11 CM) did not yield [M+H]^+^ ions. A plausible explanation is that protonated molecules of this oligoester type are very prone to dissociation in the gas phase. Since [M+H]^+^ ions may be regarded as the most important from an analytical point of view, their absence may make the identification of these oligoesters more difficult in comparison to the other oligoester types.

**Figure 4:**
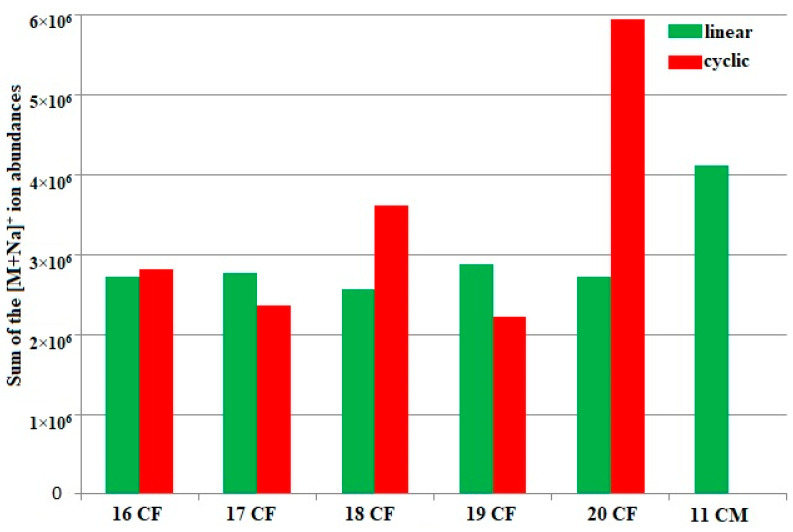
The relative content of linear and cyclic oligoesters in the analyzed samples.

Cone voltage increase leads to gas-phase ion dissociation, which further brings a decrease in the parent ion abundance. However, with increasing cone voltage, the parent ion abundances (peak intensities) may also increase since at higher cone voltage, more ions reach the high vacuum region of the mass spectrometer. In the latter case, we noted this for [M+Na]^+^ ions. In other words, the cone voltage increase from 30 V to 50 V did not lead to the gas-phase dissociation of [M+Na]^+^ ions, and it increased the respective peak intensities, which is clearly evidenced in the mass spectra obtained (Figure 3 and Figure S5–S8). Therefore, the sum of the abundances of the [M+Na]^+^ ions was used to compare (semi-quantitatively) the relative content of linear and cyclic oligoesters in the analyzed samples. As shown in Figure 4, the highest amount of cyclic oligoesters was found in sample 20 CF, which contained NPG and AA/iPA as comonomers.

**Figure 5:**
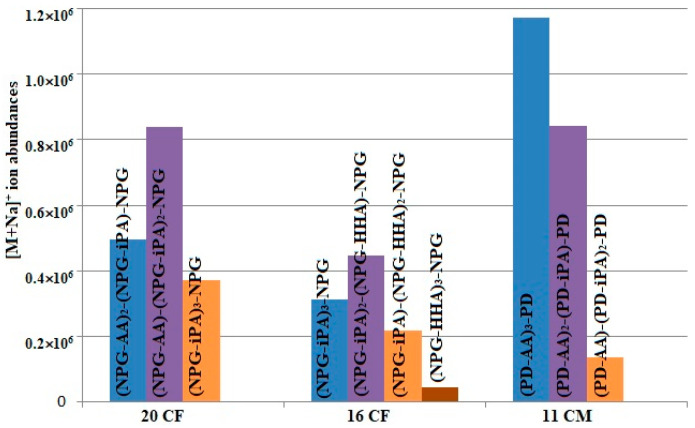
The exemplary relative contributions of individual cooligoesters.

In samples 16 CF, 17 CF, 18 CF, and 19 CF, which contained compounds composed of NPG and iPA/HHA as comonomers, the relative contents of linear and cyclic oligoesters were comparable. It has been already established that cyclic oligoesters are more toxic than linear ones since the former are usually assessed as Cramer III substances, whereas the latter is usually assessed as Cramer I substances [6,10,17,20]. Therefore, polyester-based food contact materials composed of NPG and AA/iPA as comonomers can be regarded as the source of more dangerous potential migrants than the other types. On the other hand, cooligoesters consisting of PD and AA/iPA as comonomers were found to be exclusively linear (Figure 4, sample 11 CM). Furthermore, for this type of cooligoester, the most abundant were aromatic ring non-containing compounds, whereas for the other types of cooligosters, aromatic ring non-containing compounds were not detected or had minor abundances, as shown in Figure 5. Therefore, polyester-based food contact materials composed of PD and AA/iPA seem to be the safest with respect to consumer health than the others.

The detected linear cooligoesters were exclusively those containing one diol unit more than diacid units, e.g., the diol-terminated ones. Those containing one diacid unit more than diol units and those containing an equal number of diol and diacid units (e.g, the diacid-terminated ones) were not detected. Analogous results were reported by Cariou et al. [29]. It may be argued that in positive ion mode, diacid-terminated cooligoesters were not detected as they should be analyzed in negative ion mode. However, in ESI positive ion mode, carboxylic acid may also be detected on the basis of [M+H]^+^, [M+Na]^+^, or [M+H-H_2_O]^+^ ions [41,42,43]. Therefore, it is plausible that if diacid-terminated cooligoesters were present in the analyzed can-coating materials in comparable amounts to the diol-terminated ones, they would be detected.

## Figures and Tables

**Table 1:** Detected linear NPG conjugates.

Compound, M (Da); rt (min); Sample	Detected Ions
(NPG-iPA)_2_-NPG; 572; 20.8 16 CF, 17 CF; 18 CF, 19 CF;	[M+Na]^+^ *m*/*z* 595; [M+NH_4_]^+^ *m*/*z* 590; [M+H]^+^ *m*/*z* 573; [M+H-H_2_O]^+^ *m*/*z* 555; [M+H-104]^+^ *m*/*z* 469
(NPG-iPA)-(NPG-HHA)-NPG; 578; 20.5 16 CF, 17 CF, 18 CF, 19 CF	[M+Na]^+^ *m*/*z* 601; [M+NH_4_]^+^ *m*/*z* 596; [M+H]^+^ *m*/*z* 579; [M+H-H_2_O]^+^ m/z 561; [M+H-104]^+^ *m*/*z* 475
(NPG-HHA)_2_-NPG; 584; 20.2 16 CF, 17 CF, 18 CF, 19 CF,	[M+Na]^+^ *m*/*z* 607; [M+NH_4_]^+^ *m*/*z* 602; [M+H]^+^ *m*/*z* 585; [M+H-H_2_O]^+^ *m*/*z* 567; [M+H-104]^+^ *m*/*z* 481
(NPG-AA)_2_-(NPG-iPA)-NPG; 766; 22.6 20 CF	[M+Na]^+^ *m*/*z* 789; [M+NH_4_]^+^ *m*/*z* 784; [M+H]^+^ *m*/*z* 767; [M+H-H_2_O]^+^ *m*/*z* 749; [M+H-104]^+^ *m*/*z* 663
(NPG-AA)-(NPG-iPA)_2_-NPG; 786; 23.6 20 CF	[M+Na]^+^ *m*/*z* 809; [M+NH_4_]^+^ *m*/*z* 804; [M+H]^+^ *m*/*z* 787; [M+H-H_2_O]^+^ *m*/*z* 769; [M+H-104]^+^ *m*/*z* 683
(NPG-iPA)_3_-NPG; 806; 24.4 16 CF, 17 CF, 18 CF, 19 CF, 20 CF,	[M+Na]^+^ *m*/*z* 829; [M+NH_4_]^+^ *m*/*z* 824; [M+H]^+^ *m*/*z* 807; [M+H-H_2_O]^+^ *m*/*z* 789; [M+H-104]^+^ *m*/*z* 703
(NPG-iPA)_2_-(NPG-HHA)-NPG; 812; 24.4, 16 CF, 17 CF, 18 CF, 19 CF,	[M+Na]^+^ *m*/*z* 835; [M+NH_4_]^+^ *m*/*z* 830; [M+H]^+^ *m*/*z* 813; [M+H-H_2_O]^+^ *m*/*z* 795; [M+H-104]^+^ *m*/*z* 709
(NPG-iPA)-(NPG-HHA)_2_-NPG; 818; 24.2 16 CF, 17 CF, 18 CF, 19 CF,	[M+Na]^+^ *m*/*z* 841; [M+NH_4_]^+^ *m*/*z* 836; [M+H]^+^ *m*/*z* 819; [M+H-H_2_O]^+^ *m*/*z* 801; [M+H-104]^+^ *m*/*z* 715
(NPG-HHA)_3_-NPG; 824; 24.0, 16 CF, 17 CF, 18 CF, 19 CF,	[M+Na]^+^ *m*/*z* 847; [M+NH_4_]^+^ *m*/*z* 842; [M+H]^+^ *m*/*z* 825; [M+H-H_2_O]^+^ *m*/*z* 807; [M+H-104]^+^ *m*/*z* 721
(NPG-AA)_3_-(NPG-iPA)-NPG; 980; 24.83 20 CF	[M+Na]^+^ *m*/*z* 1003; [M+NH_4_]^+^ *m*/*z* 998; [M+H]^+^ *m*/*z* 981; [M+H-H_2_O]^+^ *m*/*z* 963; [M+H-104]^+^ *m*/*z* 877
(NPG-AA)_2_-(NPG-iPA)_2_-NPG; 1000; 25.6 20 CF	[M+Na]^+^ *m*/*z* 1023; [M+NH_4_]^+^ *m*/*z* 1018; [M+H]^+^ *m*/*z* 1001; [M+H-H_2_O]^+^ *m*/*z* 983; [M+H-104]^+^ *m*/*z* 897
(NPG-AA)-(NPG-iPA)_3_-NPG; 1020; 26.3; 20 CF	[M+Na]^+^ *m*/*z* 1043; [M+NH_4_]^+^ *m*/*z* 1038; [M+H]^+^ *m*/*z* 1021; [M+H-H_2_O]^+^ *m*/*z* 1003; [M+H-104]^+^ *m*/*z* 917
(NPG-iPA)_4_-NPG; 1040; 26.9 17 CF, 19 CF, 20 CF,	[M+Na]^+^ *m*/*z* 1063; [M+NH_4_]^+^ *m*/*z* 1058; [M+H]^+^ *m*/*z* 1041; [M+H-H_2_O]^+^ *m*/*z* 1023; [M+H-104]^+^ *m*/*z* 937
(NPG-iPA)_3_-(NPG-HHA)-NPG; 1046; 27.0; 17 CF, 19 CF	[M+Na]^+^ *m*/*z* 1069; [M+NH_4_]^+^ *m*/*z* 1064; [M+H]^+^ *m*/*z* 1047; [M+H-H_2_O]^+^ *m*/*z* 1029; [M+H-104]^+^ *m*/*z* 943
(NPG-iPA)_2_-(NPG-HHA)_2_-NPG; 1052; 26.9 17 CF, 19 CF	[M+Na]^+^ *m*/*z* 1075; [M+NH_4_]^+^ *m*/*z* 1070; [M+H]^+^ *m*/*z* 1053; [M+H-H_2_O]^+^ *m*/*z* 1035; [M+H-104]^+^ *m*/*z* 949
(NPG-iPA)-(NPG-HHA)_3_-NPG; 1058; 26.8 17 CF, 19 CF	[M+Na]^+^ *m*/*z* 1081; [M+NH_4_]^+^ *m*/*z* 1076; [M+H]^+^ *m*/*z* 1059; [M+H-H_2_O]^+^ *m*/*z* 1041; [M+H-104]^+^ *m*/*z* 955

**Table 2:** Detected linear PD conjugates in sample 11 CM.

Compound, M (Da); rt (min)	Detected Ions
(PD-AA)_3_-PD; 634; 15.6	[M+Na]^+^ *m*/*z* 657; [M+NH_4_]^+^ *m*/*z* 652; [M+H-H_2_O]^+^ *m*/*z* 617; [M+H-76]^+^ *m*/*z* 559
(PD-AA)_2_-(PD-iPA)-PD; 654; 16.8	[M+Na]^+^ *m*/*z* 677; [M+NH_4_]^+^ *m*/*z* 672; [M+H-H_2_O]^+^ *m*/*z* 637; [M+H-76]^+^ *m*/*z* 579
(PD-AA)-(PD-iPA)_2_-PD; 674; 17.3	[M+Na]^+^ *m*/*z* 697; [M+NH_4_]^+^ *m*/*z* 692; [M+H-H_2_O]^+^ *m*/*z* 657; [M+H-76]^+^ *m*/*z* 599
(PD-AA)_4_-PD; 820; 18.2	[M+Na]^+^ *m*/*z* 843; [M+NH_4_]^+^ *m*/*z* 838; [M+H-H_2_O]^+^ *m*/*z* 803; [M+H-76]^+^ *m*/*z* 745
(PD-AA)_3_-(PD-iPA)-PD; 840; 18.6	[M+Na]^+^ *m*/*z* 863; [M+NH_4_]^+^ *m*/*z* 858; [M+H-H_2_O]^+^ *m*/*z* 823; [M+H-76]^+^ *m*/*z* 765
(PD-AA)_2_-(PD-iPA)_2_-PD; 860; 19.1	[M+Na]^+^ *m*/*z* 883; [M+NH_4_]^+^ *m*/*z* 878; [M+H-H_2_O]^+^ *m*/*z* 843; [M+H-76]^+^ *m*/*z* 785
(PD-AA)_5_-PD; 1006; 19.9	[M+Na]^+^ *m*/*z* 1029; [M+NH_4_]^+^ *m*/*z* 1024; [M+H-H_2_O]^+^ *m*/*z* 989; [M+H-76]^+^ *m*/*z* 931
(PD-AA)_4_-(PD-iPA)-PD; 1026; 20.1	[M+Na]^+^ *m*/*z* 1049; [M+NH_4_]^+^ *m*/*z* 1044; [M+H-H_2_O]^+^ *m*/*z* 1009; [M+H-76]^+^ *m*/*z* 951
(PD-AA)_3_-(PD-iPA)_2_-PD; 1046; 20.4	[M+Na]^+^ *m*/*z* 1069; [M+NH_4_]^+^ *m*/*z* 1064; [M+H-H_2_O]^+^ *m*/*z* 1029; [M+H-76]^+^ *m*/*z* 971

**Table 3:** Detected cyclic NPG conjugates.

Compound, M (Da); rt (min); Sample	Detected Ions
(NPG-AA)_3_; 642; 23.6 20 CF	[M+Na]^+^ *m*/*z* 665; [M+NH_4_]^+^ *m*/*z* 660; [M+H]^+^ *m*/*z* 643; [M+H-86]^+^ *m*/*z* 557; [M+H-214]^+^ *m*/*z* 429
(NPG-AA)_2_-(NPG-iPA); 662; 24.8 20 CF	[M+Na]^+^ *m*/*z* 685; [M+NH_4_]^+^ *m*/*z* 680; [M+H]^+^ *m*/*z* 663; [M+H-86]^+^ *m*/*z* 577; [M+H-214]^+^ *m*/*z* 449
(NPG-AA)-(NPG-iPA)_2_; 682; 25.8 20 CF	[M+Na]^+^ *m*/*z* 705; [M+NH_4_]^+^ *m*/*z* 700; [M+H]^+^ *m*/*z* 683; [M+H-86]^+^ *m*/*z* 597; [M+H-214]^+^ *m*/*z* 469
(NPG-iPA)_3_; 702; 26.2 16 CF, 17 CF, 18 CF, 19 CF, 20 CF	[M+Na]^+^ *m*/*z* 725; [M+NH_4_]^+^ *m*/*z* 720; [M+H]^+^ *m*/*z* 703; [M+H-86]^+^ *m*/*z* 617; [M+H-234]^+^ *m*/*z* 469
(NPG-iPA)_2_-(NPG-HHA); 708; 26.1 16 CF, 17 CF, 18 CF, 19 CF	[M+Na]^+^ *m*/*z* 731; [M+NH_4_]^+^ *m*/*z* 726; [M+H]^+^ *m*/*z* 709; [M+H-86]^+^ *m*/*z* 623; [M+H-234]^+^ *m*/*z* 475
(NPG-iPA)-(NPG-HHA)_2_; 714; 26.2 16 CF, 17 CF, 18 CF, 19 CF	[M+Na]^+^ *m*/*z* 737; [M+NH_4_]^+^ *m*/*z* 732; [M+H]^+^ *m*/*z* 715; [M+H-234]^+^ *m*/*z* 481
(NPG-HHA)_3_; 720; 26.1 16 CF, 17 CF, 18 CF, 19 CF	[M+Na]^+^ *m*/*z* 743; [M+NH_4_]^+^ *m*/*z* 738; [M+H]^+^ *m*/*z* 721
(NPG-AA)_3_-(NPG-iPA); 876; 26.8 20 CF	[M+Na]^+^ *m*/*z* 899; [M+NH_4_]^+^ *m*/*z* 894; [M+H]^+^ *m*/*z* 877; [M+H-86]^+^ *m*/*z* 791; [M+H-214]^+^ *m*/*z* 663
(NPG-AA)_2_-(NPG-iPA)_2_; 896; 27.4 20 CF	[M+Na]^+^ *m*/*z* 919; [M+NH_4_]^+^ *m*/*z* 914; [M+H]^+^ *m*/*z* 897; [M+H-86]^+^ *m*/*z* 811; [M+H-214]^+^ *m*/*z* 683 [M+H-234]^+^ *m*/*z* 663
(NPG-AA)-(NPG-iPA)_3_; 916; 28.0 20 CF	[M+Na]^+^ *m*/*z* 939; [M+NH_4_]^+^ *m*/*z* 934; [M+H]^+^ *m*/*z* 917; [M+H-86]^+^ *m*/*z* 831; [M+H-234]^+^ *m*/*z* 683
(NPG-iPA)_4_; 936; 28.5 16 CF, 17 CF, 18 CF, 19 CF, 20 CF	[M+Na]^+^ *m*/*z* 959; [M+NH_4_]^+^ *m*/*z* 954; [M+H]^+^ *m*/*z* 937; [M+H-86]^+^ *m*/*z* 851; [M+H-234]^+^ *m*/*z* 703
(NPG-iPA)_3_-(NPG-HHA); 942; 28.6 16 CF, 17 CF, 18 CF, 19 CF	[M+Na]^+^ *m*/*z* 965; [M+NH_4_]^+^ *m*/*z* 960; [M+H]^+^ *m*/*z* 943; [M+H-86]^+^ *m*/*z* 857; [M+H-234]^+^ *m*/*z* 709
(NPG-iPA)_2_-(NPG-HHA)_2_; 948; 28.7 16 CF, 17 CF, 18 CF, 19 CF	[M+Na]^+^ *m*/*z* 971; [M+NH_4_]^+^ *m*/*z* 966; [M+H]^+^ *m*/*z* 949;
(NPG-iPA)-(NPG-HHA)_3_; 954; 28.9 16 CF, 17 CF, 18 CF, 19 CF	[M+Na]^+^ *m*/*z* 977; [M+NH_4_]^+^ *m*/*z* 972; [M+H]^+^ *m*/*z* 955;

## Data Availability

The raw data supporting the conclusions of this article will be made available by the authors on request.

## Supplementary Materials

The following supporting information can be downloaded at: https://www.mdpi.com/article/10.3390/ma17112771/s1. Table S1: List of canned food samples; Figure S1: Exemplary single ion chromatograms of [M+Na]^+^ ions derived from linear (NPG-iPA/HHA)_4_-NPG cooligoesters (sample 19 CF); Figure S2: Exemplary single ion chromatograms of [M+Na]^+^ ions derived from cyclic (NPG-iPA/HHA)_4_ cooligoesters (sample 16 CF); Figure S3: Exemplary single ion chromatograms of [M+Na]^+^ ions derived from linear (PD-AA/iPA)_3_-PD cooligoesters (sample 11 CM); Figure S4: Exemplary single ion chromatograms of [M+Na]^+^ ions derived from cyclic (NPG-AA/iPA)_4_ cooligoesters (sample 20 CF); Figure S5: ESI mass spectra of linear (NPG-AA)-(NPG-iPA)_2_-NPG oligoester and its plausible structure (sample 20 CF); Figure S6: ESI mass spectra of cyclic (NPG-AA)_2_-(NPG-iPA)_2_ oligoester and its plausible structure (sample 20 CF); Figure S7: ESI mass spectra of linear (PD-AA)_3_-PD oligoester and its plausible structure (sample 11 CM); Figure S8: ESI mass spectra of linear (PD-AA)_4_-(PD-iPA)-PD oligoester and its plausible structure (sample 11 CM); Figure S9: Single ion chromatograms derived from (NPG-AA)_3_-(NPG-iPA)-NPG (sample 20 CF).
