# Supplementary material for: Oligoester Identification in the Inner Coatings of Metallic Cans by High-Pressure Liquid Chromatography–Mass Spectrometry with Cone Voltage-Induced Fragmentation
Source: Materials (Basel). 2024 Jun 6;17(11):2771. doi: 10.3390/ma17112771 (PMC11173705; doi:10.3390/ma17112771)
Supplement: Supplementary file 1 [file materials-17-02771-s001.zip › materials-3044559-supplementary.pdf]

Supplementary Material

Oligoester Identification in the Inner Coatings of Metallic Cans by High-Pressure Liquid Chromatography–Mass Spectrometry with Cone Voltage-Induced Fragmentation

Monika Beszterda-Buszczak[1] and Rafał Frański[2,*]

[1] Department of Food Biochemistry and Analysis, Poznań University of Life Sciences, Mazowiecka 48, 60-623 Poznań, Poland

[2] Faculty of Chemistry, Adam Mickiewicz University, Uniwersytetu Poznańskiego 8, 61-614 Poznań, Poland

Table S1. List of canned food samples.

Food	Food category	Sample acronim
Diced tomatoes	Vegetables	1 CV
Green peas		2 CV
Mixed vegetables		3 CV
Red beans		4 CV
Sweet corn		5 CV
Apricot halves	Fruit	6 CF
Fruit coctail		7 CF
Pear halves		8 CF
Pineapple slices		9 CF
Sliced peaches		10 CF
Ham with jelly	Meat	11 CM
Goulash		12 CM
Chicken liver pâté		13 CM
Duck liver pâté		14 CM
Pork knuckle		15 CM
Herring fillets with raisins	Fish	16 CF
Herring fillets in vegetable oil		17 CF
Mackerel fillets		18 CF
Sprat in spicy sauce		19 CF
Tuna steak in olive oil		20 CF

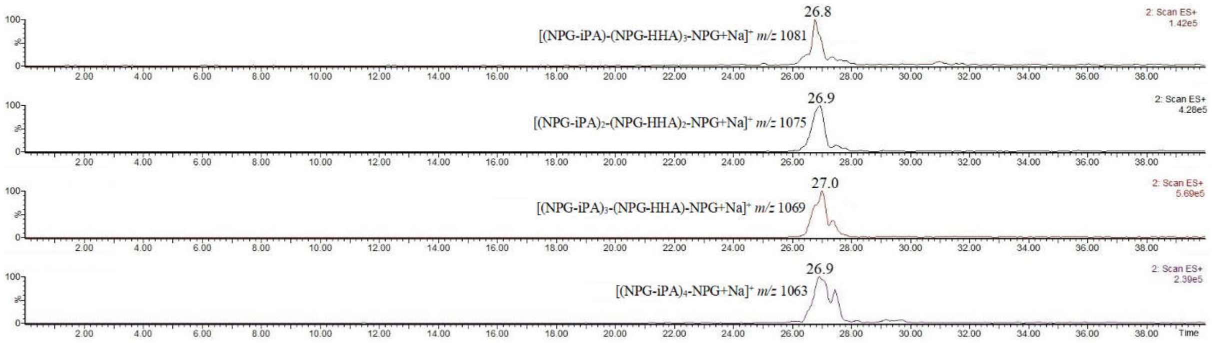

Figure S1. Exemplary single ion chromatograms of $[M+Na]^+$ ions derived from linear (NPG-iPA/HHA)$_4$-NPG cooligoesters (sample 19 CF).

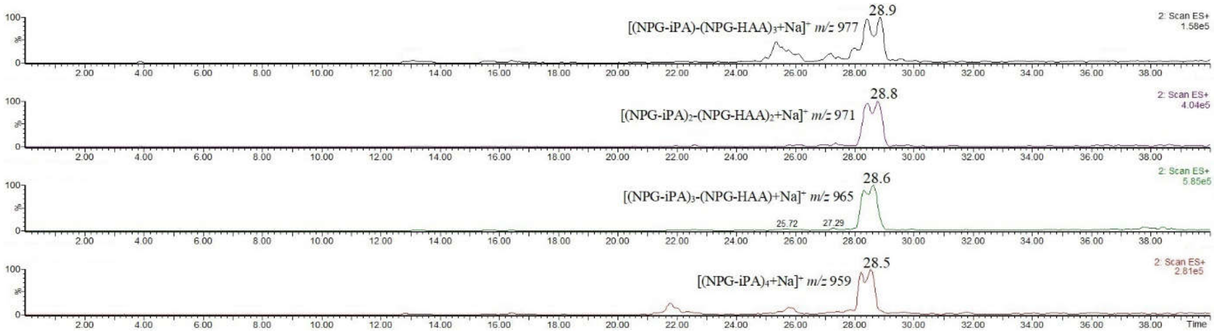

Figure S2. Exemplary single ion chromatograms of $[M+Na]^+$ ions derived from cyclic (NPG-iPA/HHA)$_4$ cooligoesters (sample 16 CF).

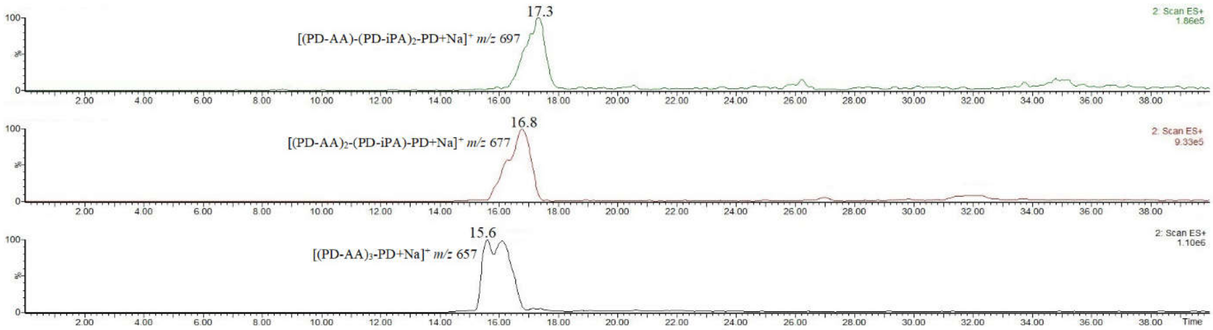

Figure S3. Exemplary single ion chromatograms of $[M+Na]^+$ ions derived from linear (PD-AA/iPA)$_3$-PD cooligoesters (sample 11 CM).

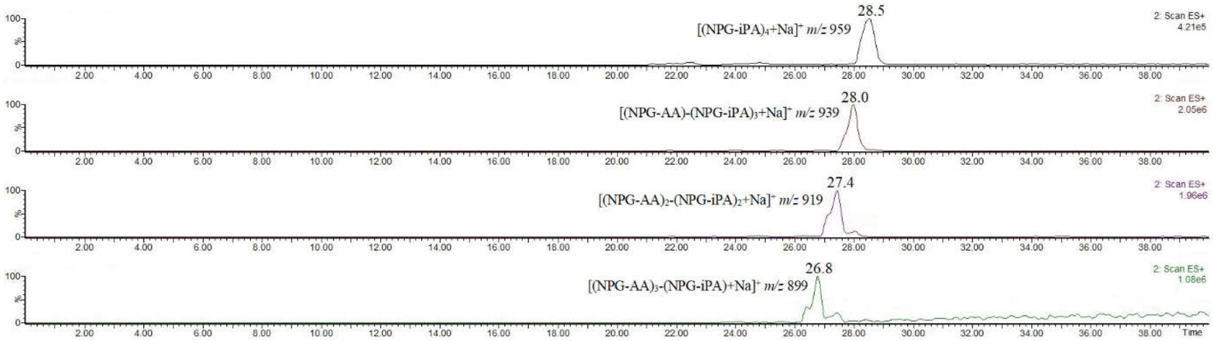

Figure S4. Exemplary single ion chromatograms of $[M+Na]^+$ ions derived from cyclic (NPG-AA/iPA)$_4$ cooligoesters (sample 20 CF).

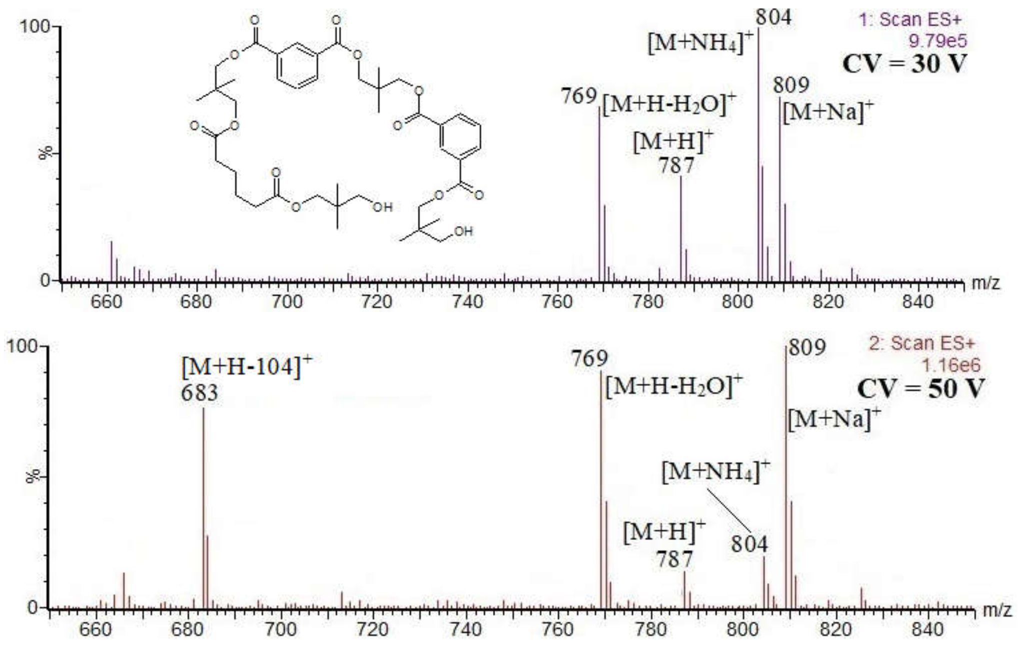

Figure S5. ESI mass spectra of linear (NPG-AA)-(NPG-iPA)$_2$-NPG oligoester and its plausible structure (sample 20 CF).

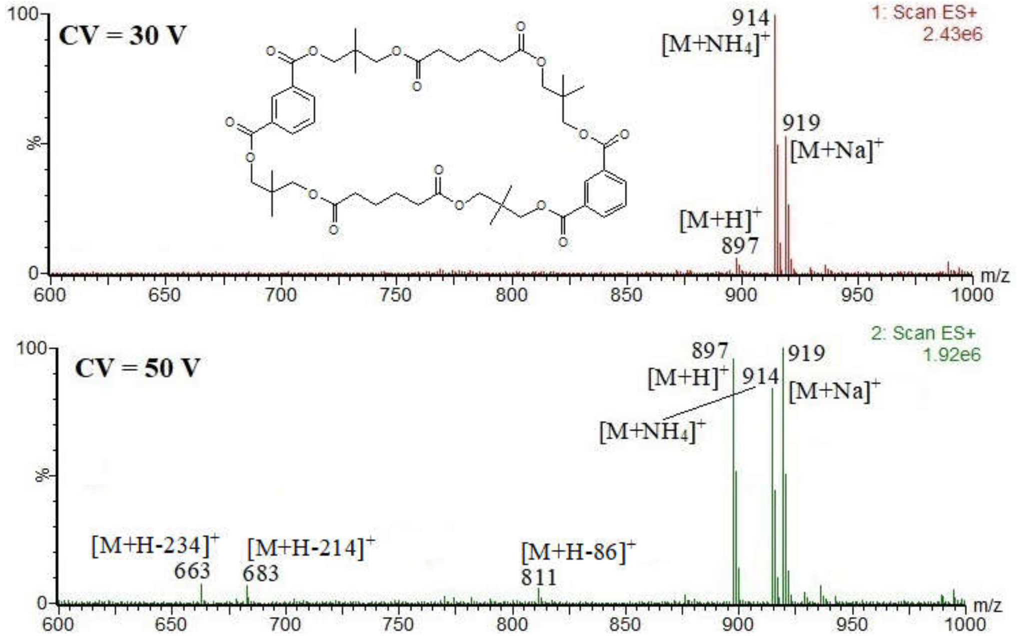

Figure S6. ESI mass spectra of cyclic (NPG-AA)$_2$-(NPG-iPA)$_2$ oligoester and its plausible structure (sample 20 CF).

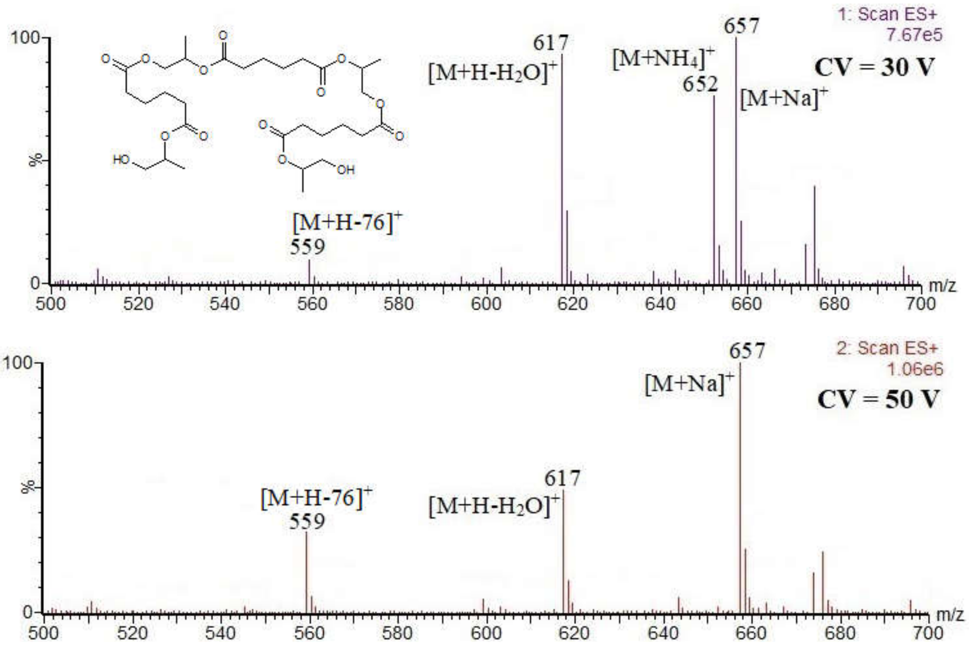

Figure S7. ESI mass spectra of linear $(PD\text{-}AA)_3$-PD oligoester and its plausible structure (sample 11 CM).

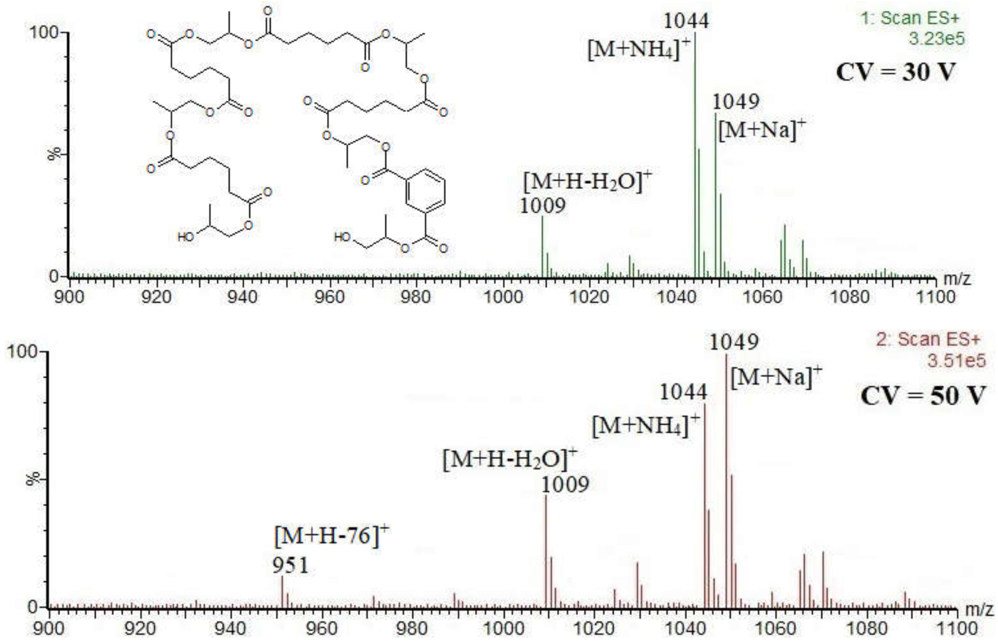

Figure S8. ESI mass spectra of linear $(PD\text{-}AA)_4$-(PD-iPA)-PD oligoester and its plausible structure (sample 11 CM).

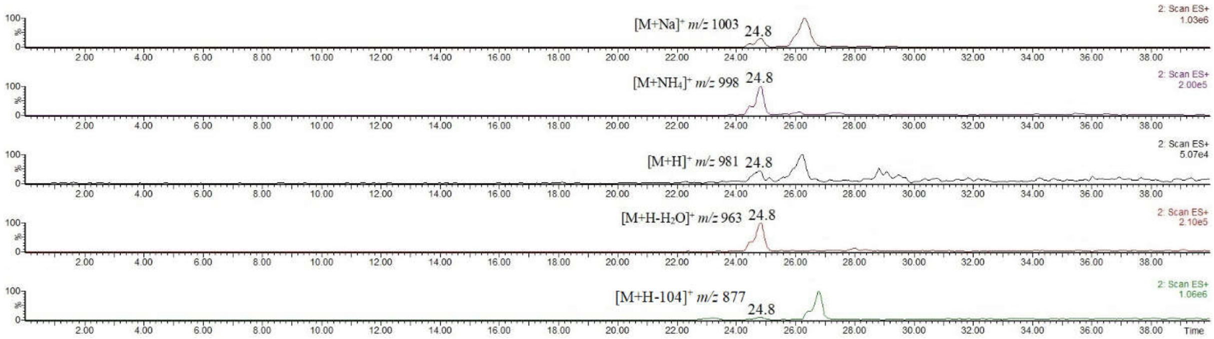

Figure S9. Single ion chromatograms derived from (NPG-AA)$_3$-(NPG-iPA)-NPG (sample 20 CF).
